# Quantitative analysis of diffuse electron scattering in the lithium-ion battery cathode material Li_1.2_Ni_0.13_Mn_0.54_Co_0.13_O_2_

**DOI:** 10.1107/S2052252522007746

**Published:** 2022-09-01

**Authors:** Romy Poppe, Daphne Vandemeulebroucke, Reinhard B. Neder, Joke Hadermann

**Affiliations:** a University of Antwerp, Groenenborgerlaan 171, Antwerp B-2020, Belgium; b Friedrich-Alexander-Universität Erlangen-Nürnberg, Staudtstrasse 3, D-91058 Erlangen, Germany; Ben-Gurion University of the Negev, Israel

**Keywords:** 3D electron diffraction, diffuse scattering, lithium-ion batteries, stacking faults, twinning, dynamical effects, scanning transmission electron microscopy, STEM

## Abstract

In contrast to perfectly periodic crystals, materials with short-range order produce diffraction patterns that contain both Bragg reflections and diffuse scattering. To understand the influence of short-range order on material properties, current research focuses increasingly on the analysis of diffuse scattering. This article verifies the possibility to refine the short-range order parameters in submicrometre-sized crystals from diffuse scattering in single-crystal electron diffraction data. The approach was demonstrated on Li_1.2_Ni_0.13_Mn_0.54_Co_0.13_O_2_, which is a state-of-the-art cathode material for lithium-ion batteries. The intensity distribution of the 1D diffuse scattering in the electron diffraction patterns of Li_1.2_Ni_0.13_Mn_0.54_Co_0.13_O_2_ depends on the number of stacking faults and twins in the crystal. A model of the disorder in Li_1.2_Ni_0.13_Mn_0.54_Co_0.13_O_2_ was developed and both the stacking fault probability and the percentage of the different twins in the crystal were refined using an evolutionary algorithm in *DISCUS*. The approach was applied on reciprocal space sections reconstructed from 3D electron diffraction data since they exhibit less dynamical effects compared with in-zone electron diffraction patterns. A good agreement was achieved between the calculated and the experimental intensity distribution of the diffuse scattering. The short-range order parameters in submicrometre-sized crystals can thus successfully be refined from the diffuse scattering in single-crystal electron diffraction data using an evolutionary algorithm in *DISCUS*.

## Abstract 2

The stacking fault probability in submicrometre-sized crystals of Li_1.2_Ni_0.13_Mn_0.54_Co_0.13_O_2_ was refined through the quantitative analysis of diffuse electron scattering.

## 1. Introduction

Structure solution and refinement are widely used to determine the average crystal structure of materials by analysis of the Bragg reflections. However, the properties of many materials depend on deviations from the average crystal structure, also called ‘short-range order’. In contrast to perfectly periodic crystals, materials with short-range order produce diffraction patterns that contain both Bragg reflections and diffuse scattering (Welberry, 2010 ▸; Sawa, 2016 ▸).

Li-rich Mn-rich layered oxides (Li_1+*x*_ *M*_1−*x*_O_2_, with *M* = Ni, Mn, Co) are promising cathode materials for lithium-ion batteries due to their high specific capacity of more than 250 mA h g^−1^. However, their commercialization is still hampered by a significant capacity and voltage decay on cycling (Liu, Wang, Zhang *et al.*, 2016 ▸; Xiang *et al.*, 2017 ▸; Pimenta *et al.*, 2017 ▸; Nayak *et al.*, 2018 ▸). Where the contributions of transition metal (TM) ion migration and spinel domain formation to the capacity and voltage decay have been extensively studied (Sathiya *et al.*, 2013 ▸; Gu *et al.*, 2013 ▸; Chen & Islam, 2016 ▸; Shimoda *et al.*, 2017 ▸; Kleiner *et al.*, 2018 ▸), the contribution of the number of stacking faults has so far only been investigated by Serrano-Sevillano *et al.* (2018 ▸). They refined the average number of stacking faults in Li_2_MnO_3_ from the diffuse scattering in powder X-ray diffraction data using the program *FAULTS* (Casas-Cabanas *et al.*, 2006 ▸) and found that the voltage decay is smaller for crystals with a larger number of stacking faults. However, further research is needed to determine a possible correlation between the number of stacking faults and the voltage decay on cycling.

Because cathode materials for lithium-ion batteries consist of submicrometre-sized crystals (Paulus *et al.*, 2020 ▸), they are too small to be investigated with single-crystal X-ray diffraction and single-crystal neutron diffraction. Therefore, single-crystal electron diffraction was used here to quantify the number of stacking faults in Li_1.2_Ni_0.13_Mn_0.54_Co_0.13_O_2_ (Li-rich Mn-rich NMC, denoted hereafter as LMR-NMC). An advantage of single-crystal electron diffraction compared with powder X-ray diffraction is that allows us to quantify the change in the number of stacking faults during cycling in an electrochemical cell using *in situ* 3D electron diffraction (3DED) (Karakulina *et al.*, 2018 ▸). This would not be possible with *in situ* transmission electron microscopy (TEM) imaging, since the spatial resolution in an electrochemical cell filled with a liquid electrolyte is too low to see the stacking faults in LMR-NMC (Hadermann & Abakumov, 2019 ▸).

In most studies on the diffuse scattering in single-crystal electron diffraction data, the diffuse scattering has been interpreted in a qualitative way, by comparing the diffuse scattering in experimental data to the diffuse scattering in simulated data (Withers *et al.*, 2003 ▸, 2004 ▸; Fujii *et al.*, 2007 ▸; Goodwin *et al.*, 2007 ▸; Brázda *et al.*, 2016 ▸; Zhao *et al.*, 2017 ▸; Neagu & Tai, 2017 ▸). A quantitative analysis of the diffuse scattering in single-crystal electron diffraction data has only recently been reported by Krysiak *et al.* (2018 ▸, 2020 ▸), who employed a fitting procedure to a series of simulated data to determine the number of stacking faults. In this article, we verify the possibility to refine the number of stacking faults from the diffuse scattering in single-crystal electron diffraction data using a differential evolutionary algorithm in *DISCUS* (Proffen & Neder, 1997 ▸). To our knowledge, the evolutionary algorithm has so far only been applied on powder X-ray diffraction data (Neder & Korsunskiy, 2005 ▸; Bürgi *et al.*, 2005 ▸; Neder *et al.*, 2007 ▸; Page *et al.*, 2011 ▸; Sławiński *et al.*, 2014 ▸, 2016 ▸, 2018 ▸) and single-crystal X-ray diffraction data (Weber & Bürgi, 2002 ▸).

## 2. Experimental

### 2.1. Synthesis

The Li_1.2_Ni_0.13_Mn_0.54_Co_0.13_O_2_ (LMR-NMC) powder was prepared by a carbonate co-precipitation method followed by calcination. The details of the synthesis were published by Paulus *et al.* (2020 ▸).

### 2.2. Transmission electron microscopy

The samples were characterized using high-angle annular dark-field scanning transmission electron microscopy (HAADF-STEM), selected area electron diffraction (SAED) and 3DED. To prepare the samples, the powder was dispersed in ethanol using an ultrasonic bath. A few droplets of the suspension were deposited on a copper grid covered with an amorphous carbon film.

The in-zone SAED patterns were acquired with an FEI Tecnai G2 electron microscope operated at 200 kV and recorded using an FEI Eagle 2k CCD camera (2048 × 2048 pixels with 16-bit dynamic range). In-zone precession electron diffraction (PED) patterns were acquired with a precession angle of 1°, using a Digistar precession device from Nanomegas.

The HAADF-STEM images and the 3DED data were acquired with an aberration-corrected cubed FEI Titan 80–300 electron microscope operated at 300 kV. For the 3DED data, a nanocrystal was tilted around the goniometer axis of the electron microscope and 2D electron diffraction patterns were acquired. The electron diffraction patterns were collected in an automated way, using an in-house developed script, and were acquired with a GATAN US1000XP CCD camera (4096 × 4096 pixels with 16-bit dynamic range). A 20 µm C2 aperture was used to produce a parallel beam of 550 nm in diameter on the sample. The crystal was recentred inside the aperture every 3° so that it was entirely illuminated during the whole data collection. The experiment was performed with a Fischione tomography holder with a tilt range of ±75°. Electron diffraction patterns were acquired in steps of 0.2°. The exposure time for each electron diffraction pattern was set to 1 s. The *PETS2* software package (Palatinus *et al.*, 2019 ▸) was used to combine the electron diffraction patterns and to reconstruct the 3D reciprocal lattice. Reciprocal space sections were reconstructed with a pixel size of 0.007 Å^−1^ and a slab thickness of 0.014 Å^−1^. No symmetry averaging was applied in the reciprocal space sections.

Since diffuse scattering is some orders of magnitude weaker than the Bragg reflections, background subtraction is important for the quantitative analysis of diffuse scattering. The background of the SAED patterns as well as the 3DED data were subtracted using *PETS2*. A Matlab script was developed to extract the intensity profile of the diffuse streaks and convert it to input for *DISCUS* (Proffen & Neder, 1997 ▸).

### 2.3. Disorder modelling and diffraction simulations

The *DISCUS* software package was used to build a model of the stacking faults and twin domains in LMR-NMC and to calculate the corresponding single-crystal electron diffraction patterns as well as the intensity distribution of the 1D diffuse scattering.

*DISCUS* calculates the intensities in reciprocal space according to the standard formula for kinematic scattering *I*(*hkl*) = *F*(*hkl*)F*(*hkl*) (Neder & Proffen, 2008 ▸) where the structure factor for electron scattering

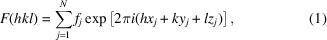

where *N* is the number of atoms in the crystal; *f_j_* is the atomic form factor of atom *j*; and *x_j_*, *y_j_* and *z_j_* are the fractional coordinates of atom *j*. The sum is calculated for each *hkl* point in reciprocal space. For finite-sized crystals, the truncation of the Fourier transform gives rise to additional, unwanted intensities in the simulated diffraction pattern. To avoid these finite-size effects, the number of pixels in the simulated diffraction pattern was chosen according to Neder & Proffen (2008 ▸):

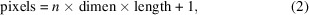

where pixels is the number of pixels along a certain direction in reciprocal space, 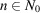 , dimen is the number of unit cells along the corresponding direction in real space and length is the length of the reciprocal space segment. All the intensity profiles were calculated for a crystal that consists of 4000 layers of 64 unit cells. The intensities were averaged over 1000 calculations to create a smooth intensity distribution.

To refine the stacking fault probability and the percentage of the different twins in the crystal, the model of the LMR-NMC crystal with stacking faults and twin domains was implemented in a differential evolutionary algorithm (Price *et al.*, 2005 ▸) in *DISCUS*. Table S1 of the supporting information shows the refinement parameters and the control parameters that were used for the refinement. To speed up the calculation, the 28 children were calculated in parallel. The refinement of the short-range order parameters took 3 days for 50 generations, using 28 cores in parallel. The refinement script can be found in the supporting information.

## 3. Results and discussion

### 3.1. Description of the disorder

**Figure 1:**
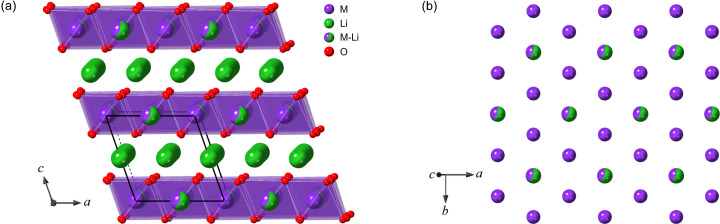
(*a*) Crystal structure of layered LMR-NMC observed along the [010] direction of the monoclinic *C*2/*m* unit cell (indicated in black). Purple octahedra represent MO_6_ octahedra, with *M* = Ni, Mn, Co. (*b*) Honeycomb ordering of the lithium-rich positions in the TM layers. Green, purple and red spheres represent lithium atoms, TM atoms and oxygen atoms, respectively.

The crystal structure of Li_1.2_Ni_0.13_Mn_0.54_Co_0.13_O_2_ (LMR-NMC) consists of alternating layers of oxygen atoms, layers of lithium atoms, and layers that contain both TM atoms and lithium atoms [see Fig. 1 ▸(*a*)]. The monoclinic *C*2/*m* unit cell (Jarvis *et al.*, 2012 ▸; Koga *et al.*, 2012 ▸; Shukla *et al.*, 2015 ▸) is indicated in black. Fig. 1 ▸(*b*) shows the honeycomb ordering of the lithium-rich positions in the TM layers, also called honeycomb layers (Bréger *et al.*, 2005 ▸; Lei *et al.*, 2009 ▸; Jarvis *et al.*, 2011 ▸).

**Figure 2:**
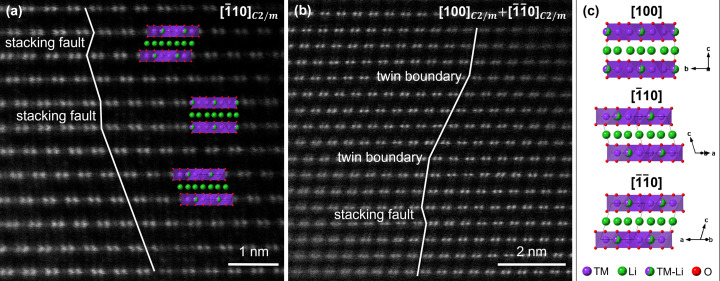
(*a*) and (*b*) HAADF-STEM images of LMR-NMC. The bright dots correspond to columns of TM atoms, whereas the less bright dots correspond to columns that contain both lithium atoms and TM atoms. The lines in (*a*) and (*b*) show the lateral displacements of the honeycomb layers. At the scale of a few unit-cell repetitions, stacking faults and twin boundaries can be observed. (*c*) The two stacking faults indicated in (*a*) are equivalent to the *C*2/*m* unit cell observed along the [100] and [1 10] directions. Green, purple and red spheres represent lithium atoms, TM atoms and oxygen atoms, respectively.

Fig. 2 shows two HAADF-STEM images of LMR-NMC. The honeycomb ordering of the lithium-rich positions in the TM layers manifests itself as pairs of 0.14 nm separated bright dots with less bright dots in between. As the intensity of the atom columns in the HAADF-STEM images is proportional to the atomic number of the element (*I* ≈ *Z* ^2^), the bright dots correspond to atom columns of TM atoms, whereas the less bright dots correspond to atom columns that contain both lithium atoms and TM atoms. The atom columns that contain lithium atoms and oxygen atoms are too weak to be observed (Paulus *et al.*, 2020 ▸).

Fig. 2 ▸(*a*) shows the HAADF-STEM image of an LMR-NMC crystal observed along the [110] direction of the monoclinic *C*2/*m* unit cell. In a crystal without stacking faults, all lithium-rich positions would lie in rows parallel to the *c* axis of the monoclinic *C*2/*m* unit cell. However, in reality, stacking faults occur due to lateral displacements of the honeycomb layers. If the stacking direction of adjacent layers is the same over several unit cells, but not over the whole crystal, then these adjacent layers will form twin domains. The difference between stacking faults and twin domains is clarified in the HAADF-STEM image in Fig. 2 ▸(*b*) in which the twin boundaries are indicated. In the following, we thus make a distinction between twin domains as groups of adjacent layers with the same stacking direction, and stacking faults as single layers with a different stacking direction.

The twin domains indicated in Fig. 2 ▸(*b*) are rotation twins with the threefold twin axis [103] (Riekehr *et al.*, 2016 ▸). The twin matrices for a rotation of 120 and 240° around [103] are

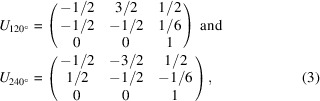

respectively. Application of both twin matrices on the [100] zone axis results in the [110] and [1 10] zone axes. Fig. 2 ▸(*c*) illustrates that the two stacking faults indicated in Fig. 2 ▸(*a*) are equivalent to the *C*2/*m* unit cell observed along the [100] and [1 10] directions.

### 3.2. Experimental data

**Figure 3:**
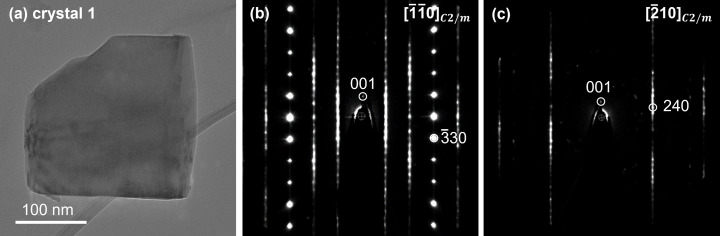
(*a*) LMR-NMC crystal and the (*b*) [1 10] and (*c*) [210] reciprocal space sections reconstructed from a 3DED series acquired.

Fig. 3 ▸(*a*) shows an image of a single LMR-NMC crystal on which we acquired 3DED data (this crystal will herein be denoted crystal 1). The reciprocal lattice was indexed with the *C*2/*m* unit cell, of which the cell parameters are given in Table S2. Figs. 3 ▸(*b*) and 3(*c*) show the reconstructed [1 10] and [210] reciprocal space sections, respectively. The diffuse streaks along the c* direction can be attributed to stacking faults. The intensity maxima along the diffuse streaks are caused by rotation twins with the threefold twin axis [103]. Fig. S1 of the supporting information shows the effect of threefold rotation twins on the [001], [010], [100], [110] and [1 10] reciprocal space sections.

To see if the orientation of the crystal in the sample holder affects the intensity profile of the diffuse streaks in the reciprocal space sections, three 3DED series were acquired on the same crystal (denoted hereafter crystal 2), with the same settings (beam size, intensity, exposure time), but with a different orientation of the grid in the sample holder. The reciprocal lattice of all three 3DED series was indexed with the *C*2/*m* unit cell; the cell parameters are given in Table S2.

**Figure 4:**
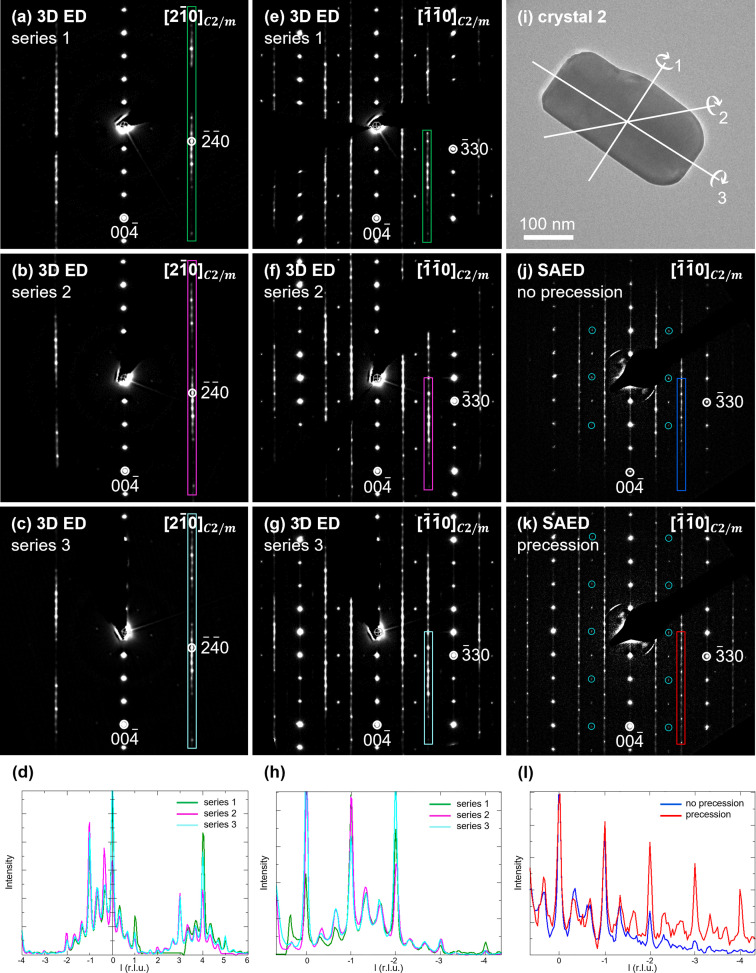
(*a*)–(*c*) [210] and (*e*)–(*g*) [1 10] reciprocal space sections reconstructed from three 3DED series acquired on the same crystal, but with the grid rotated over 45° (series 2) and 90° (series 3) clockwise compared with series 1. (*d*) and (*h*) Intensity profiles of the diffuse streaks indicated in (*a*)–(*c*) and (*e*)–(*g*). (*i*) LMR-NMC crystal on which all 3DED series and SAED patterns have been acquired. The rotation axes corresponding to series 1, 2 and 3 are indicated. (*j*) [1 10] SAED pattern, which corresponds to the same orientation as the reciprocal space sections in (*e*)–(*g*). (*k*) The same SAED pattern but acquired with a precession angle of 1°. The reflections circled in blue in (*j*) and (*k*) can be attributed to dynamical diffraction. (*l*) Intensity profile of the diffuse streaks indicated in (*j*) and (*k*).

Figs. 4 ▸(*a*)–4(*c*) and 4 ▸(*e*)–4(*g*) show the [210] and the [1 10] reciprocal space sections reconstructed from series 1, 2 and 3, respectively. For series 2, the grid was rotated 45° clockwise compared with series 1. For series 3, the grid was rotated 90° clockwise compared with series 1. The [001], [010], [100], [110] and [1 10] reciprocal space sections are shown in Fig. S2. The intensity profiles of the diffuse streaks indicated in Figs. 4 ▸(*a*)–4(*c*) and 4 ▸(*e*)–4(*g*) are shown in Figs. 4 ▸(*d*) and Fig. 4 ▸(*h*), respectively. The intensities of the strong peaks are different for series 1, 2 and 3, which imply that the orientation of the crystal in the sample holder affects the intensity profile of the diffuse streaks. From the dynamical theory of diffraction, we know that the reflection intensities depend on the orientation and thickness of the crystal (Palatinus *et al.*, 2015 ▸). Since we acquired data on a rod-shaped crystal, the crystal thickness may change during the collection of the 3DED series, depending on the rotation axis. The rotation axes corresponding to series 1, 2 and 3 are indicated in Fig. 4 ▸(*i*). For series 1 the crystal thickness is larger at high tilt angles than at low tilt angles (±340 nm versus ±175 nm). For series 3 the crystal thickness stays approximately the same during the whole tilt series (±175 nm). Consequently, the amount of dynamical scattering will be larger for series 1 than for series 3. The crystal thickness was estimated by taking images of the crystal at different alpha angles. More details can be found in the supporting information.

The reciprocal space sections in Figs. 3 ▸ and 4 ▸ were reconstructed without frame scaling (frame intensity scale equal to 1 for all frames) since the frame scales calculated by *PETS2* are less reliable for low-symmetry crystal systems. To calculate the frame scales, *PETS2* matches the intensities of symmetry equivalent reflections. The presence of rotation twins with the threefold twin axis [103] reduces the Laue class from 2/*m* to 1. For Laue class 1, the only symmetry equivalent reflections are on the same frames or on the immediately neighbouring frames, making the frame scaling less reliable.

Thermal vibration of the atoms in a crystal gives rise to a diffuse background in electron diffraction patterns (Muller *et al.*, 2001 ▸). To verify whether this diffuse background would persist after background removal by *PETS2*, two 3DED series were acquired on the same LMR-NMC crystal (crystal 2): one with and one without an energy filter. The energy filter was used to block inelastically scattered electrons with an energy loss of more than 10 eV. The effect of using an energy filter was only small (Fig. S3), therefore all further 3DED series have been acquired without energy filtering.

To compare the difference between reciprocal space sections reconstructed from 3DED series and SAED patterns, all SAED patterns and 3DED series in Fig. 4 ▸ were acquired on the same crystal. Fig. 4 ▸(*j*) shows the [1 10] SAED pattern, which corresponds to the same orientation as the reciprocal space sections in Figs. 4 ▸(*e*)–4(*g*). Fig. 4 ▸(*k*) shows the same SAED pattern but acquired with a precession angle of 1°. The intensity profile of the diffuse streaks indicated in Figs. 4 ▸(*j*) and 4(*k*) are shown in Fig. 4 ▸(*l*).

In precession electron diffraction (PED), the electron beam is tilted away from the optical axis of the microscope and rotated on the surface of a cone (Vincent & Midgley, 1994 ▸). The dynamical effects are reduced due to the off-axis beam inclinations because fewer beams are simultaneously excited (Oleynikov *et al.*, 2007 ▸). The intensities in a PED pattern are also less sensitive to crystal imperfections like thickness variations or crystal bending (Palatinus *et al.*, 2015 ▸). Consequently, the intensities further away from the central beam are more intense [Fig. 4 ▸(*l*)].

Comparing the in-zone PED pattern with the reciprocal space sections reconstructed from 3DED data acquired on the same crystal shows that several reflections in the PED pattern are solely due to dynamical diffraction (reflections indicated by the blue circles). Besides, the increase in intensity between *l* = −1 and *l* = −2 in the intensity profiles of the diffuse streaks in the reciprocal space sections [Fig. 4 ▸(*h*)] is not visible in the intensity profile of the diffuse streak in the PED pattern [Fig. 4 ▸(*l*)]. Since reciprocal space sections are reconstructed from a set of off-zone electron diffraction patterns, they exhibit less dynamical effects compared with in-zone electron diffraction patterns. A higher precession angle could further reduce the dynamical scattering. However, for LMR-NMC a precession angle higher than 1° led to overlap with reflections from higher-order Laue zones.

### 3.3. Additional types of twinning

The weak reflections between the diffuse streaks in the [1 10] reciprocal space sections in Fig. 4 ▸ are not present in the calculated [1 10] electron diffraction pattern in Fig. S1. At first glance, these additional reflections could be due to the presence of the spinel phase (Quintelier *et al.*, 2021 ▸). However, careful inspection of the 3D reciprocal lattice shows that they are due to rotation twins with the fourfold twin axis [323]. These rotation twins are domains with a different orientation of the Li- and TM-layers (Riekehr *et al.*, 2016 ▸; Liu, Wang, Ding *et al.*, 2016 ▸; Jarvis *et al.*, 2014 ▸). Fig. S4 gives an overview of the zones that overlap with the [001], [010], [100], [110] and [1 10] zones as a result of both rotation twinning with the threefold twin axis [103] and rotation twinning with the fourfold twin axis [323]. The weak reflections between the diffuse streaks in the [1 10] reciprocal space sections in Fig. 4 ▸ are thus due to the overlap of the [1 10] zone axis with the [3 1 6], [316] and [001] zone axes.

Inspection of the 3D reciprocal lattice of 20 different LMR-NMC crystals shows that all crystals have rotation twins with the threefold twin axis [103], while only 7 of the 20 crystals have rotation twins with the fourfold twin axis [323] (domains with a different orientation of the Li- and TM-layers). Reflection splitting in the [010] reciprocal space section (Fig. S5) corresponds to reflection twins with the mirror plane (001) (Abakumov *et al.*, 2021 ▸; Yin *et al.*, 2020 ▸). Reflection twins were present in 5 of the 20 investigated crystals. Since both rotation twins with the fourfold twin axis [323] and reflection twins with the mirror plane (001) affect the intensity profile of the diffuse streaks, the refinement will be applied on crystal 1, which only has rotation twins with the threefold twin axis [103].

### 3.4. Model of the disorder

To simulate an LMR-NMC crystal with stacking faults and rotation twins with the threefold twin axis [103], we created a stack of several layers. Each layer is a slab of the *C*2/*m* crystal structure with a thickness of one *C*2/*m* unit cell along the *c* direction. As the lithium-rich positions in subsequent honeycomb layers can shift relative to each other by **c**, 1/3**b** + **c**, or 1/2**a** + 1/6**b** + **c**, we defined the translation vectors *M_ij_* between two adjacent layers *i* and *j* by

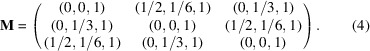

For instance, the translation vector *M*_23_ = (1/2, 1/6, 1) on the second row and the third column means that a layer of type 3 is shifted by 1/2**a** + 1/6**b** + **c** relative to the previous layer when that layer is of type 2. In our model, all layer types are identical but undergo a different translation, so each layer type gets a different numbering.

As stacking faults (single layers with a different translation vector) may occur in each twin domain (a group of adjacent layers with the same translation vector), we defined the transition probability matrices for the [100], [110] and [1 10] twin domain by

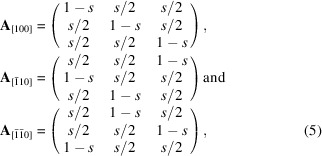

where *A_ij_* is the probability for one of the translation vectors *M_ij_* to be chosen and *s* is the stacking fault probability (0 ≤ *s* ≤ 1), considered identical for the [100], [110] and [1 10] twins. The layers of the [110] and [1 10] twin domains were rotated by 120 and 240° around [103], respectively.

**Figure 5:**
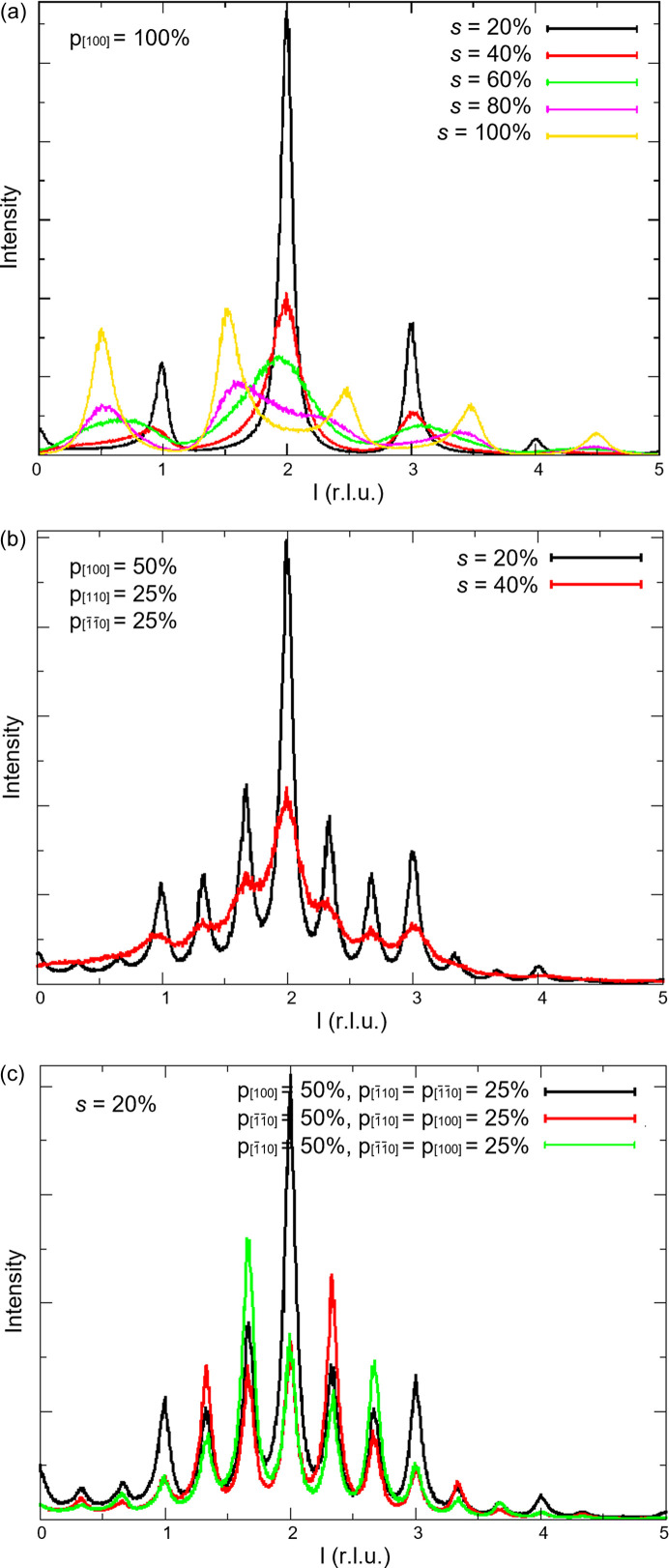
Intensity profile of the 04*l* diffuse streak (*a*) for a crystal that consists of only one twin (*p*_[100]_ = 100%) and for a stacking fault probability of 20, 40, 60, 80 and 100%; (*b*) for a crystal that consists of all [100], [110] and [1 10] twin domains (*p*_[100]_ = 50% and 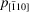 = 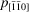 = 25%) and for a stacking fault probability of 20 and 40%; (*c*) for different percentages of the [100], [110] and [1 10] twin domains. *s* is the stacking fault probability and *p*_[100]_ and 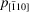 are the percentages of the [100] and the [110] twins in the crystal, respectively.

Fig. 5 ▸(*a*) shows the intensity profile of the 04*l* diffuse streak for increasing stacking fault probability. When the stacking fault probability increases from 0 to 60%, the discrete intensity maxima at integer *l* values merge together. From a stacking fault probability of 80% onward, intensity peaks with maxima at half-integer *l* values appear. The reason is that, for a stacking fault probability of 100%, only two possible shifts can occur, which results again in a more periodic stacking of the layers. The intensity profiles in Fig. 5 ▸(*a*) were calculated for a crystal that consists of only one twin, whereas the intensity profiles in Fig. 5 ▸(*b*) were calculated for a crystal that consists of all [100], [110] and [1 10] twin domains. Rotation twinning with the threefold twin axis [103] causes additional intensity maxima along the diffuse streaks at *l* = *n* + 1/3 and/or *l* = *n* + 2/3. In the following, the percentage of the [100], [110] and [1 10] twin axes in the crystal will be denoted by *p*_[100]_, 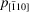 and 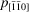 , respectively. The intensity profiles in Fig. 5 ▸(*b*) were calculated for *p*_[100]_ = 50% and 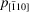 = 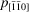 = 25%. To illustrate the effect of different percentages of the [100], [110] and [1 10] twin domains on the intensity profile of the 04*l* diffuse streak, the intensity profiles in Fig. 5 ▸(*c*) were calculated for different values of *p*_[100]_, 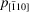 and 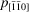 (black curve: *p*_[100]_ = 50%, 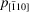 = 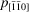 = 25%; red curve: 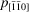 = 50%, *p*_[100]_ = 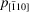 = 25%; green curve: 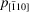 = 50%, *p*_[100]_ = 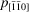 = 25%).

### 3.5. Refinement of the disorder

As discussed in the previous section, the intensity distribution of the diffuse streaks depends on both the stacking fault probability *s* and the percentage of the [100], [110] and [1 10] twins in the crystal denoted by *p*_[100]_, 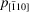 and 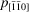 . Since 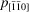 = 1 − *p*_[100]_ − 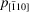 , it was sufficient to refine *s*, *p*_[100]_ and 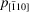 .

Refining the atomic coordinates and occupancies from our 3DED data in *Jana2020* (Petrícek *et al.*, 2014 ▸) was not successful, probably because of the combination of stacking faults, rotation twins with the threefold twin axis [103] and dynamical effects. Therefore, we used the atomic coordinates, atomic displacement parameters, occupancies and cell parameters of Li_1.2_Ni_0.15_Mn_0.55_Co_0.1_O_2_ determined by Mohanty *et al.* (2013 ▸) (ICSD entry 237940). Since the Ni/Mn/Co ratio in Li_1.2_Ni_0.15_Mn_0.55_Co_0.1_O_2_ is only slightly different from the Ni/Mn/Co ratio in Li_1.2_Ni_0.13_Mn_0.54_Co_0.13_O_2_ (LMR-NMC), the effect on the atomic coordinates and atomic displacement parameters will be negligible. To decrease the refinement time, we used the integer occupancies in Table S3 (the position with most lithium was considered fully occupied by lithium, the other position was considered fully occupied by manganese) instead of the occupancies determined by Mohanty *et al.* (2013 ▸). This simplification of the model has a negligible effect on the intensity profile (Fig. S6). The refinement algorithm calculates the diffuse scattering from a crystal that consists of 2000 layers of one unit cell (when using integer occupancies, the number of unit cells in one layer has no influence on the intensity profile). To create a smooth intensity distribution, the algorithm averages the intensity profile over 100 calculations.

**Figure 6:**
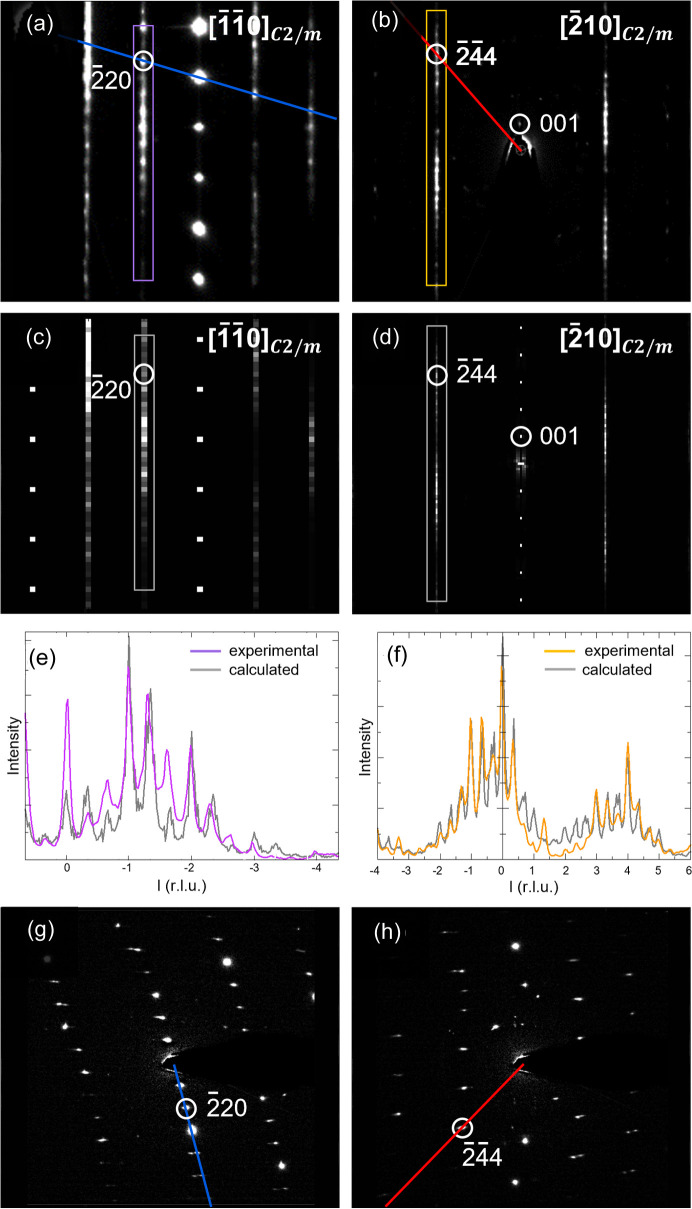
(*a*) and (*b*) Part of the [1 10] and [210] reciprocal space sections of crystal 1. (*c*) and (*d*) Electron diffraction patterns calculated for the refined short-range order parameters at generation 50. (*e*) and (*f*) Intensity profile of the diffuse streaks indicated in (*a*) and (*b*) together with the intensity profiles calculated for the refined short-range order parameters at generation 50. (*g*) and (*h*) Two electron diffraction patterns that were used to reconstruct the [1 10] and [210] sections in (*a*) and (*b*). The blue and red lines in (*g*) and (*h*) correspond to those in (*a*) and (*b*).

The refinement was applied on the intensity profile of the diffuse streaks indicated in the [1 10] and [210] reciprocal space sections in Figs. 3 ▸(*b*) and 3(*c*) [details in Figs. 6 ▸(*a*) and 6(*b*)]. Figs. S7 and S8 show the evolution of the *R* value and the short-range order parameters for the first 50 generations. The refined short-range order parameters and the *R* value at generation 50 are listed in Table 1 ▸. The electron diffraction patterns in Figs. 6 ▸(*c*) and 6(*d*) were calculated for these refined short-range order parameters, for a crystal that consists of 2000 layers of 100 unit cells. The width of the Bragg reflections and the diffuse streaks in the calculated patterns is inversely proportional to the number of unit cells in one layer.

The agreement between the experimental and the calculated intensity profiles is better for the diffuse streak indicated in the [210] section than for the diffuse streak indicated in the [1 10] section [Figs. 6(*e*) and 6(*f*)]. Since *DISCUS* calculates the intensity profiles according to the standard formula for kinematic scattering, the intensity differences between the experimental and the calculated intensity profile are likely the result of dynamical effects. The 220 reflection, for example, is much stronger in the experimental than in the calculated [1 10] section [peak at *l* = 0 in Fig. 6(*e*)]. Figs. 6(*g*) and 6(*h*) show two electron diffraction patterns that were used to reconstruct the [1 10] and [210] sections in Figs. 6(*a*) and 6(*b*). In the electron diffraction pattern in Fig. 6(*g*), more reflections are simultaneously excited than in the electron diffraction pattern in Fig. 6(*h*), which explains why dynamical effects are larger for the 220 reflection than for the 2 44 reflection.

As mentioned before, no frame scaling was applied during the reconstruction of the reciprocal space sections in Figs. 6 ▸(*a*) and 6(*b*). Frame scaling corrects for variations in the crystal thickness and/or variations in the illuminated volume. Even though the whole crystal was illuminated during the collection of the 3DED data, variations in the crystal thickness might affect the intensities and consequently also the experimental intensity profile of the diffuse streaks.

Small intensity differences between the experimental and the calculated intensity profiles of the diffuse streaks can also be attributed to deviations in the atomic coordinates and occupancies. The atomic coordinates we used were refined using powder neutron diffraction data (Mohanty *et al.*, 2013 ▸). However, Mohanty and co-authors did not consider stacking faults (peak broadening) and twinning (peak overlap) in their refinement, which might affect the atomic coordinates and occupancies, and consequently also the calculated intensity profile of the diffuse streaks.

Krysiak *et al.* (2018 ▸, 2020 ▸), who previously reported a quantitative analysis of diffuse scattering in single-crystal electron diffraction data on two zeolites, assigned the small intensity differences between their experimental and calculated patterns to inelastic scattering and an insufficiently sensitive detector. Because zeolites mainly consist of light elements [O (*Z* = 8) and Si (*Z* = 14)] and dynamical effects are smaller for elements with lower atomic numbers (Gorelik *et al.*, 2019 ▸), it is likely that the intensities in their reciprocal space sections were indeed less influenced by dynamical scattering. Their 3DED data were also acquired on thinner crystals (100 nm), which also reduces the amount of dynamical scattering.

An insufficiently investigated aspect of lithium-ion battery cathode materials is the contribution of the number of stacking faults to the capacity and voltage decay. To collect data on a statistically relevant number of crystals, the collection of 3DED data can be automated (Wang *et al.*, 2019 ▸). In lithium-ion battery cathode materials, stacking faults and twins are formed during crystal growth, but the number of stacking faults may change during charging and discharging. Karakulina *et al.* (2018 ▸) showed that it is possible to collect *in situ* 3DED data on lithium-ion battery cathode materials in a liquid electrolyte during cycling in an electrochemical cell. The future aim is therefore to use this method to quantify the change in the number of stacking faults in LMR-NMC during charging and discharging.

## 4. Conclusions

As the properties of many technologically important materials are associated with the disorder that exists in their crystal structures, it is important to have methods to quantify this disorder. In this article, we verified the possibility to refine the short-range order parameters in submicrometre-sized crystals from the diffuse scattering in single-crystal electron diffraction data. The approach was demonstrated on the lithium-ion battery cathode material LMR-NMC. Both the stacking fault probability and the percentage of the different twins in the crystal were refined from the intensity distribution of the diffuse streaks using an evolutionary algorithm in *DISCUS*.

The approach was applied on reciprocal space sections reconstructed from 3DED data since they exhibited less dynamical effects compared with in-zone PED patterns. For the [210] reciprocal space section of the investigated crystal, the best agreement between the calculated and the experimental intensity distribution of the diffuse scattering was achieved for a stacking fault probability of 29 (2)% and twin percentages of *p*_[100]_ = 40 (3)%, 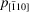 = 34 (3)% and 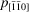 = 26 (6)%.

The experimental intensity profile depends on the orientation of the crystal in the sample holder, which can be explained by differences in the amount of dynamical diffraction. Therefore, the intensity differences between the experimental and calculated profiles are most likely due to residual dynamical effects. Other factors such as small deviations in the atomic coordinates and occupancies, inelastic scattering and an insufficiently sensitive detector could also cause small intensity differences between the experimental and calculated profiles.

## Supplementary Material

Refinement script. DOI: 10.1107/S2052252522007746/vq5002sup1.zip

Supporting information file. DOI: 10.1107/S2052252522007746/vq5002sup2.pdf

## Figures and Tables

**Table 1:** Refined short-range order parameters and the *R* value at generation 50 for the intensity profile of the diffuse streaks indicated in the [1 10] and [210] reciprocal space sections of crystal 1 *s* is the stacking fault probability, and *p*_[100]_ and 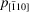 are the percentages of the [100] and [110] twins in the crystal.

Parameter	Refined value [1 10]	Refined value [210]
*s*	0.24 (2)	0.29 (2)
*p*_[100]_	0.37 (5)	0.40 (3)
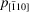	0.15 (5)	0.34 (3)
*R* value	50.3 (7)	27.8 (5)
