# Supplementary material for: Quantitative analysis of diffuse electron scattering in the lithium-ion battery cathode material Li1.2Ni0.13Mn0.54Co0.13O2
Source: IUCrJ. 2022 Sep 1;9(Pt 5):695–704. doi: 10.1107/S2052252522007746 (PMC9438490; doi:10.1107/S2052252522007746)
Supplement: Supplementary file 2 [file m-09-00695-sup2.pdf]

IUCrJ

Volume 9 (2022)

Supporting information for article:

Quantitative analysis of diffuse electron scattering in the lithium-ion battery cathode material Li1.2Ni0.13Mn0.54Co0.13O2

Romy Poppe, Daphne Vandemeulebroucke, Reinhard B. Neder and Joke Hadermann

S1. Evolutionary algorithm

The evolutionary algorithm mimics the Darwinian principle of natural evolution. The algorithm creates two groups of parameter sets (parents and children) of which the R values are calculated. The parents and the children with the lowest R values survive and will be the parents of the new generation (survival of the fittest). This procedure is repeated for a certain number of refinement cycles (generations) until the set of parameter values converges towards the actual values (Neder & Proffen, 2008).

Table S1 The refinement parameters and the control parameters used in the differential evolutionary algorithm in *DISCUS*. To prevent convergence into a local minimum instead of the global minimum, the number of children was chosen equal to the number of parents. The number of parents and the number of children were both chosen to be 28 which is the number of cores in one node on the Leibniz cluster, and which is approximately ten times the number of refined parameters.

**refinement parameters**	
number of parents	28
number of children	28
**control parameters**	
cross-over probability	0.9
scale factor	0.81
probability for a parent to be modified by a local Gaussian distribution	0
scaled difference vector	1

S2. Experimental data

Table S2 Cell parameters of the monoclinic *C2/m* unit cell of $Li_{1.2}Ni_{0.13}Mn_{0.54}Co_{0.13}O_2$ (LMR-NMC) used to index the reciprocal lattice reconstructed from the three-dimensional electron diffraction (3D ED) series in *PETS2*. For crystal 2, three 3D ED series were acquired on the same crystal, but with a different orientation of the grid in the sample holder.

	crystal 1	crystal 2		
angular range	-75° till 62°	series 1: -75° till 50°	series 2: -75° till 60°	series 3: -75° till 70°
a (Å)	5.035(2)	5.058(2)	5.0551(6)	5.084(2)
b (Å)	8.772(3)	8.780(2)	8.7697(9)	8.840(2)
c (Å)	5.107(5)	5.131(2)	5.1418(6)	5.1441(9)
α (°)	89.31(5)	89.89(2)	90.208(9)	89.88(2)
β (°)	108.71(5)	109.20(2)	109.22(1)	109.14(2)
γ (°)	90.05(3)	89.96(2)	89.978(9)	89.04(2)

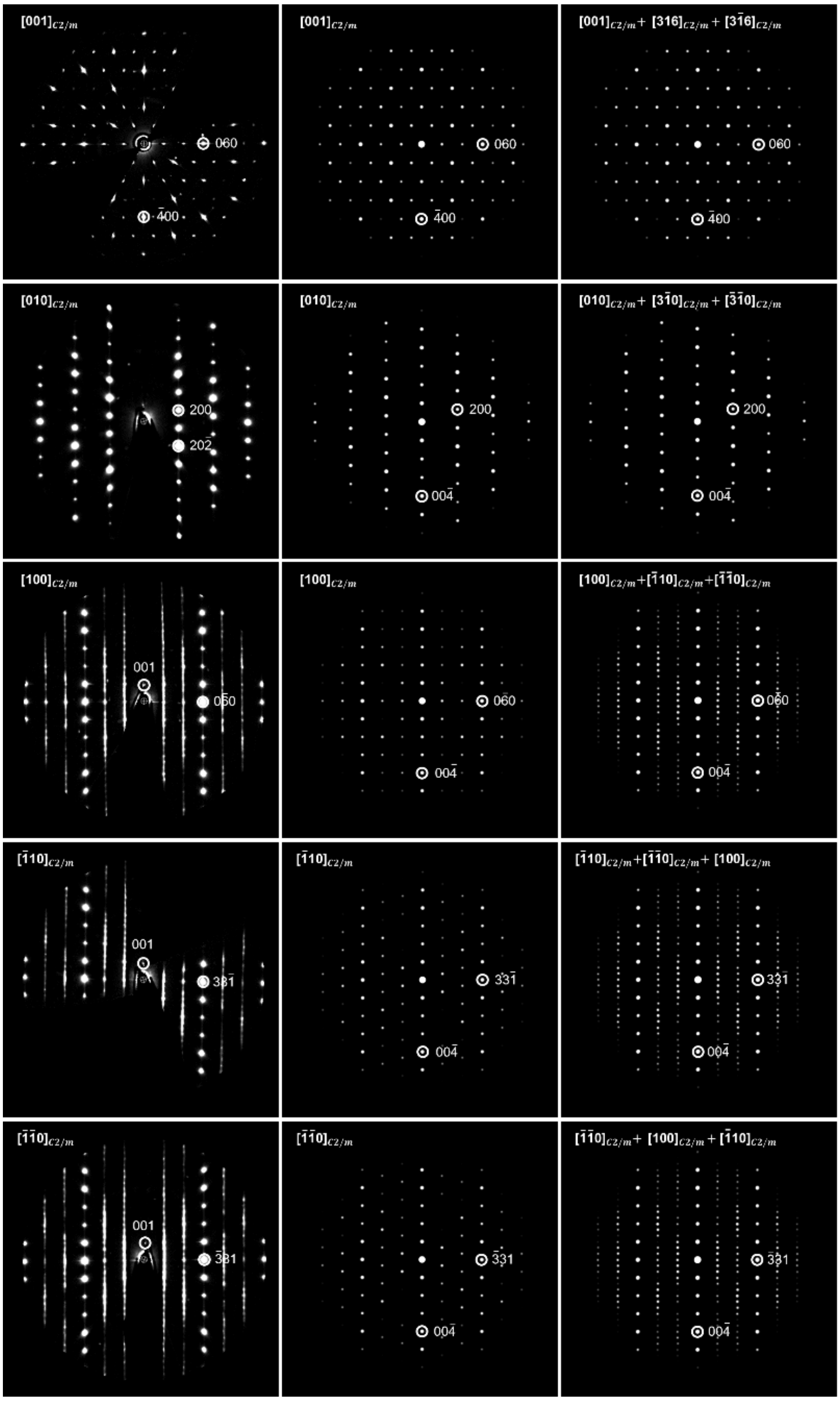

Figure S1 Column 1: [001], [010], [100], $[\bar{1}10]$ and $[\bar{1}\bar{1}0]$ reciprocal space sections reconstructed from a three-dimensional electron diffraction (3D ED) series acquired on $Li_{1.2}Ni_{0.13}Mn_{0.54}Co_{0.13}O_2$ (LMR-NMC) [crystal 1]. Column 2: calculated electron diffraction patterns for a crystal without twinning. Column 3: calculated electron diffraction patterns for a crystal with rotation twinning with 3-fold twin axis [103]. The intensity maxima along the diffuse streaks in column 1 are thus due to overlap of the reflections in the $[\bar{1}\bar{1}0]$ zone with the reflections in the $[\bar{1}10]$ and [100] zones. The electron diffraction patterns in column 2-3 were calculated in SingleCrystal.

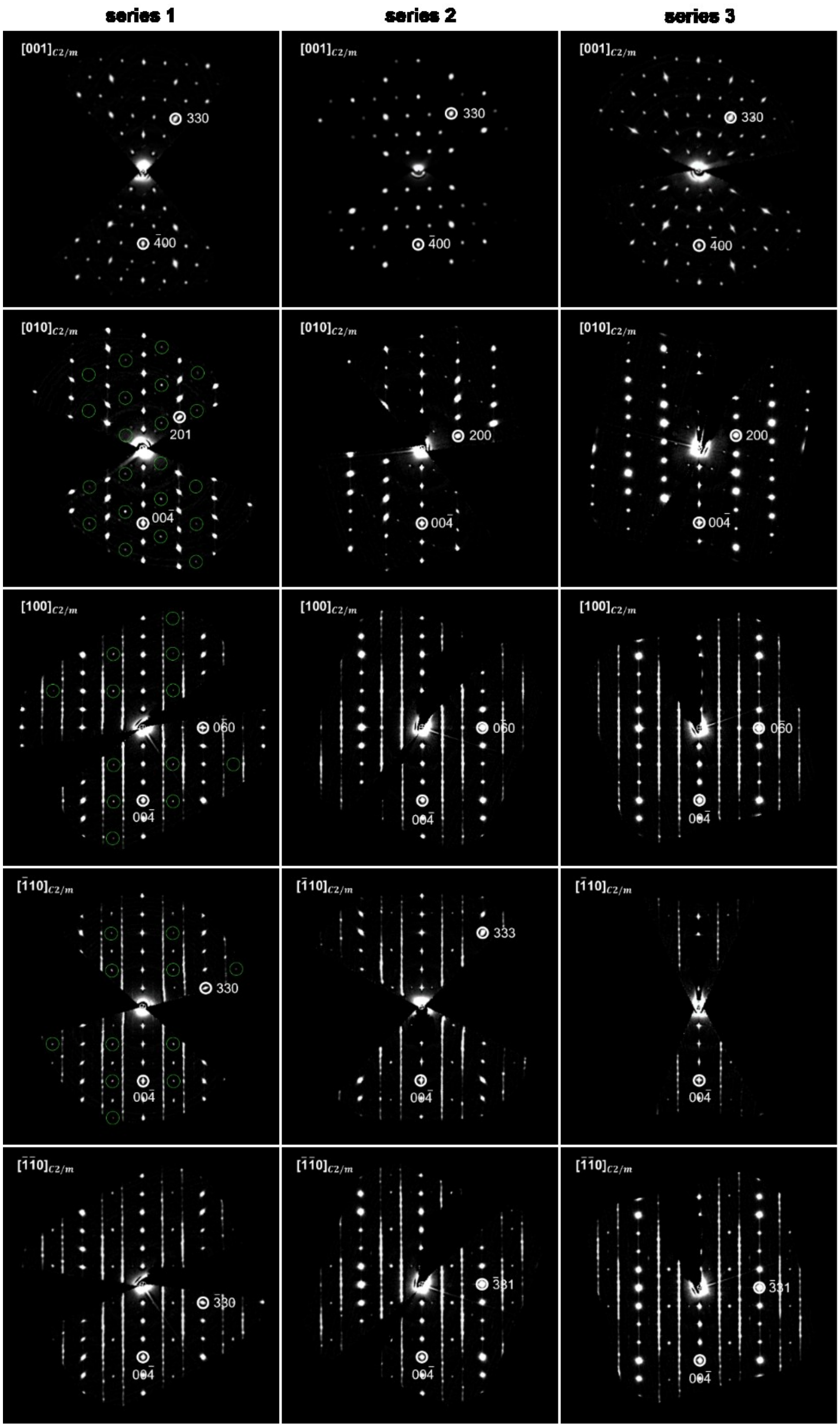

Figure S2 [001], [010], [100], $[\bar{1}10]$ and $[\bar{1}\bar{1}0]$ reciprocal space sections reconstructed from three three-dimensional electron diffraction (3D ED) series acquired on the same $Li_{1.2}Ni_{0.13}Mn_{0.54}Co_{0.13}O_2$ (LMR-NMC) crystal [crystal 2], but with the grid rotated over 45° (series 2) and 90° (series 3) clockwise compared to series 1. In the [010], [100] and $[\bar{1}10]$ sections reconstructed from series 1, additional reflections are visible (circled in green), which are absent in series 2 and 3. These reflections could be caused by twinning; however, we could not determine a twin relation that would explain all additional reflections.

S3. Crystal thickness

To estimate the crystal thickness, the grid was placed in the sample holder so that the rotation axis was parallel to the long axis of the crystal. The crystal thickness at α = 0° is then equal to the size of the short axis of the crystal at α = 90°. Images of the crystal were taken at different alpha angles (α between 0° and 70°). The size of the short axis was measured in each image and varied between 165 nm and 180 nm. Therefore, for series 3 (with the rotation axis parallel to the long axis of the crystal) the crystal thickness was assumed to stay approximately the same during the whole tilt series (175 ± 10 nm).

For series 1 (with the rotation axis parallel to the short axis of the crystal), the crystal thickness was larger at high tilt angles than at low tilt angles. To estimate the crystal thickness at α = -75° (starting angle of series 1), the size of the long axis of the crystal at α = 0° was measured (330 ± 10 nm). Assuming a rod-shaped crystal, the crystal thickness at α = -75° was estimated to be 330 ± 10 nm / cos (15°) = 340 ± 10 nm.

S4. Energy filter

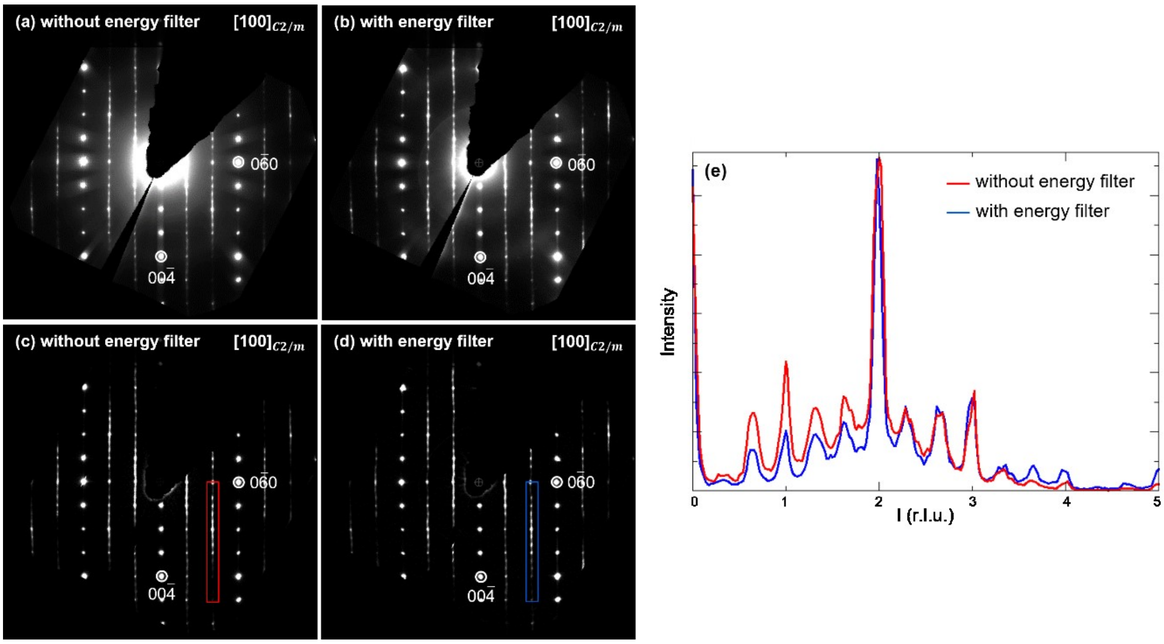

Figure S3 (a) [100] reciprocal space section reconstructed from a three-dimensional electron diffraction (3D ED) series acquired on $Li_{1.2}Ni_{0.13}Mn_{0.54}Co_{0.13}O_2$ (LMR-NMC) [crystal 2] without an energy filter. The diffuse intensity bands are due to thermal diffuse scattering. (b) [100] reciprocal space section reconstructed from a 3D ED series acquired on the same crystal but with an energy filter. The energy filter was used to block inelastically scattered electrons with an energy loss of more than 10 eV. (c-d) The reciprocal space sections in respectively (a) and (b) after background subtraction. (e) The intensity profile of the diffuse streaks indicated in (c-d).

S5. Additional types of twinning

The twin matrices for a rotation of respectively 90°, 180° and 270° around [323] are:

$$\boldsymbol{V}_{90^\circ} = \begin{pmatrix} 1/2 & 3/2 & -1/2 \\ -1/6 & 1/2 & 1/2 \\ 1 & 0 & 0 \end{pmatrix}, \boldsymbol{V}_{180^\circ} = \begin{pmatrix} -1/2 & 3/2 & 1/2 \\ 1/3 & 0 & 1/3 \\ 1/2 & 3/2 & -1/2 \end{pmatrix},$$
$$\boldsymbol{V}_{270^\circ} = \begin{pmatrix} 0 & 0 & 1 \\ 1/2 & 1/2 & -1/6 \\ -1/2 & 3/2 & 1/2 \end{pmatrix}. \quad (6)$$

Application of these three twin matrices on the $[\bar{1}\bar{1}0]$ zone axis, results in the $[\bar{6}\bar{1}\bar{3}]$, $[\bar{3}\bar{1}\bar{6}]$ and $[0\bar{1}\bar{1}]$ zone axes. Rotation twinning with 3-fold twin axis [103] causes additional overlap of the $[\bar{6}\bar{1}\bar{3}]$ zone axis with the $[01\bar{1}]$ and $[3\bar{2}\bar{3}]$ zone axes; of the $[\bar{3}\bar{1}\bar{6}]$ zone axis with the $[\bar{3}1\bar{6}]$ and $[00\bar{1}]$ zone axes; and of the $[0\bar{1}\bar{1}]$ zone axis with the $[\bar{6}1\bar{3}]$ and $[32\bar{3}]$ zone axes. Consequently, the $[\bar{1}\bar{1}0]$ zone will overlap with 11 other zones ($[\bar{1}10]$, [100], $[\bar{6}\bar{1}\bar{3}]$, $[01\bar{1}]$, $[3\bar{2}\bar{3}]$, $[\bar{3}\bar{1}\bar{6}]$, $[\bar{3}1\bar{6}]$, $[00\bar{1}]$, $[0\bar{1}\bar{1}]$, $[\bar{6}1\bar{3}]$ and $[32\bar{3}]$).

Comparing the reflections in the reciprocal space sections and in the calculated electron diffraction patterns (Fig. S4), shows that only 3 of the 4 rotation twins with 4-fold twin axis [323] are present in crystal 2. Since some of the reflections in the $[0\bar{1}\bar{1}]$ and the $[00\bar{1}]$ zone do overlap with the diffuse streaks in the $[\bar{1}\bar{1}0]$ zone, rotation twins with 4-fold twin axis [323] affect the intensity profile of the diffuse streaks.

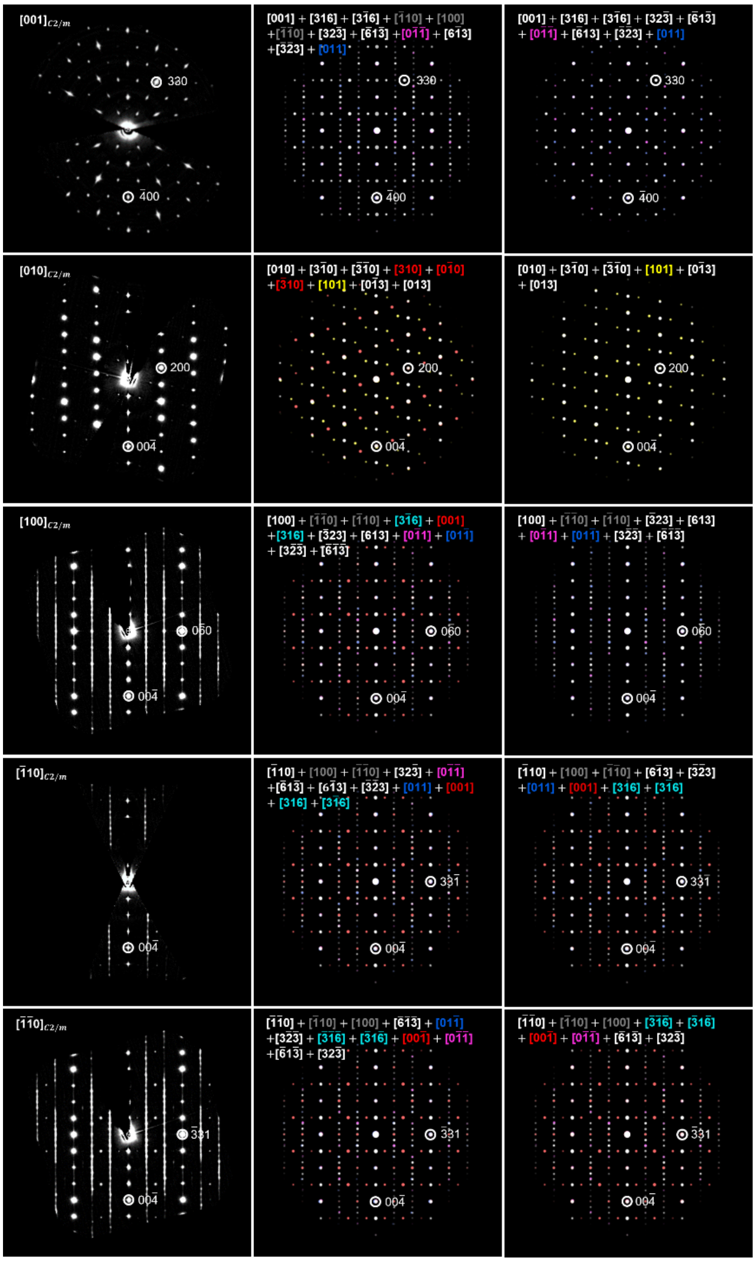

Figure S4 Column 1: [001], [010], [100], $[\bar{1}10]$ and $[\bar{1}\bar{1}0]$ reciprocal space sections reconstructed from a three-dimensional electron diffraction (3D ED) series acquired on $Li_{1.2}Ni_{0.13}Mn_{0.54}Co_{0.13}O_2$ (LMR-NMC) [crystal 2, series 3 in Fig. S2]. Column 2: calculated electron diffraction patterns for a crystal with 3 rotation twins with 3-fold twin axis [103] and 4 rotation twins with 4-fold twin axis [323]. Column 3: calculated electron diffraction patterns for a crystal with 3 rotation twins with 3-fold twin axis [103] and 3 rotation twins with 4-fold twin axis [323]. The weak reflections in the reciprocal space sections in column 1 are thus due to the presence of 2 additional twins with a different orientation of the Li- and TM-layers. The weak reflections in the [001] reciprocal space section are too weak to be observed. The electron diffraction patterns in columns 2-3 were calculated in SingleCrystal.

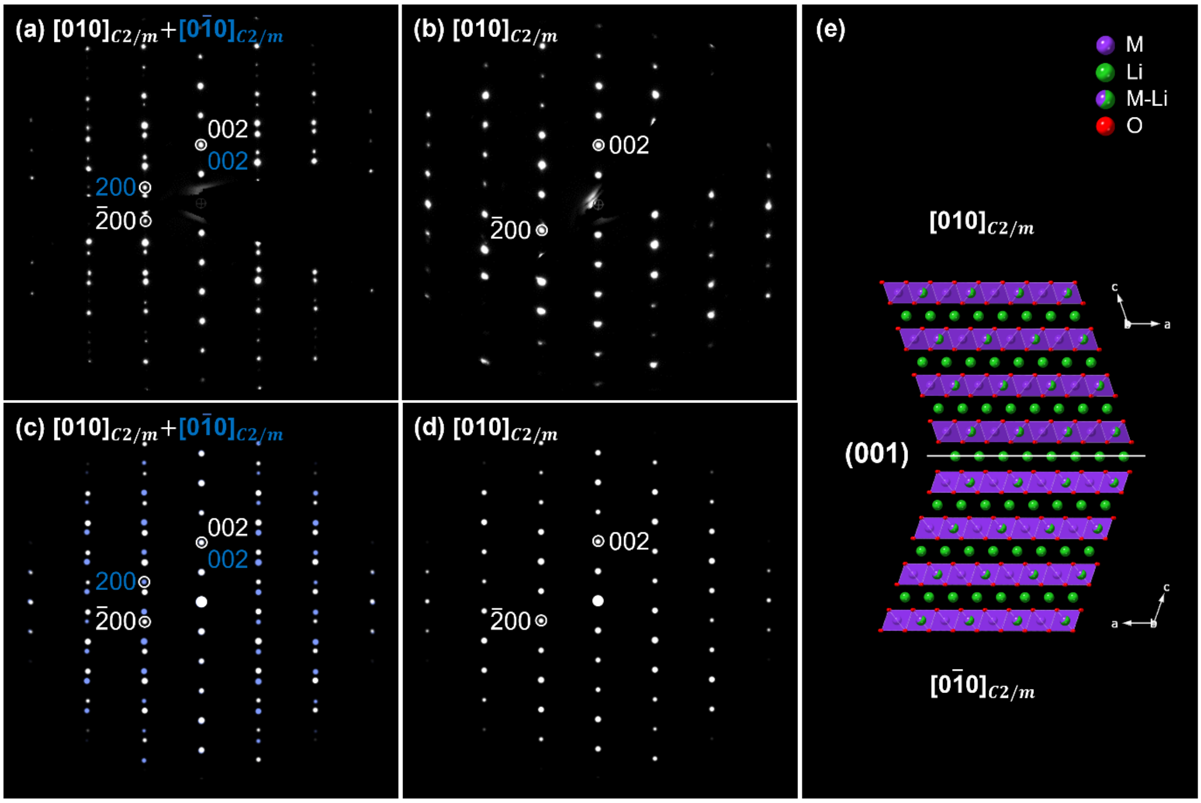

Figure S5 (a-b) Reconstructed [010] reciprocal space section of a crystal (a) with and (b) without reflection twins, indexed with the *C2/m* unit cell of $Li_{1.2}Ni_{0.13}Mn_{0.54}Co_{0.13}O_2$ (LMR-NMC). (c-d) Calculated electron diffraction patterns for a crystal (c) with and (d) without reflection twins. (e) Two reflection twins with mirror plane (001) seen along the [010] direction. The electron diffraction patterns in (c-d) were calculated in SingleCrystal. Green, purple and red spheres represent lithium atoms, transition metal atoms, and oxygen atoms, respectively.

S6. Refinement

Table S3 Column 2-4: Atomic coordinates, atomic displacement parameters and occupancies of the *C2/m* unit cell of $Li_{1.2}Ni_{0.15}Mn_{0.55}Co_{0.1}O_2$ determined by powder neutron diffraction (Mohanty *et al.*, 2013) and used for the simulation of the model in *DISCUS*. Column 5: Occupancies used for the refinement. The use of integer occupancies implies that the TM-atoms in the Li-rich positions are replaced by Li, and that all Ni (Z=28) and Co (Z=27) atoms are replaced by Mn (Z=25).

Atom type	x, y, z	U_{iso} (Å^2)	Occupancies	Integer occupancies
Li	0, 0, 0.5	0.0117(2)	0.9957(11)	1
M	0, 0, 0.5	0.0117(2)	0.0043(11)	0
Li	0, 0.6551, 0.5	0.0125(4)	0.9900(6)	1
M	0, 0.6551, 0.5	0.0125(4)	0.0100(6)	0
Li	0, 0.5, 0	0.0029(2)	0.364(1)	1
M	0, 0.5, 0	0.0029(2)	0.636(1)	0
Li	0, 0.1660, 0	0.0076(4)	0.128(1)	0
M	0, 0.1660, 0	0.0076(4)	0.872(1)	1
O	0.2226, 0, 0.2232	0.0049(4)	1	1
O	0.2539, 0.3223, 0.2256	0.0056(8)	1	1

With M = Ni, Mn, Co.

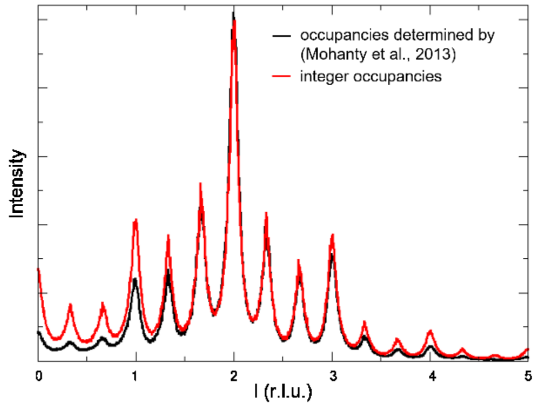

Figure S6 Intensity profile of the 04*l* diffuse streak simulated with 1) integer occupancies 2) the occupancies determined by (Mohanty *et al.*, 2013). Both profiles were calculated for a crystal with a stacking fault probability of 20% and twin percentages of $p_{[100]}$ = 50% and $p_{[\bar{1}10]} = p_{[\bar{1}\bar{1}0]}$ = 25%.

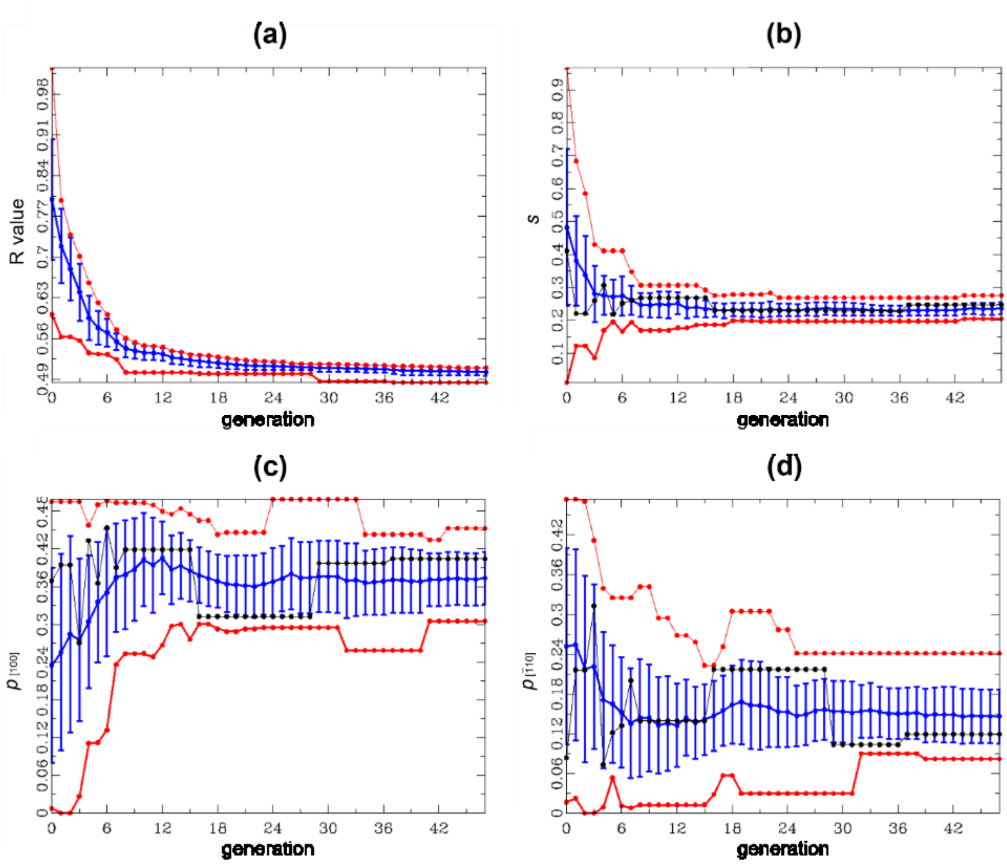

Figure S7 Refinement applied on the intensity profile of the diffuse streak indicated in the $[\bar{1}\bar{1}0]$ reciprocal space section in Fig. 6(a). Evolution of (a) the R value, (b) the stacking fault probability s, (c) the percentage $p_{[100]}$ of the $[100]$ twin and (d) the percentage $p_{[\bar{1}10]}$ of the $[\bar{1}10]$ twin as a function of the generation number. The figure shows the average (blue), smallest and highest (red) value at each generation. The value with the lowest R value at each generation is shown in black.

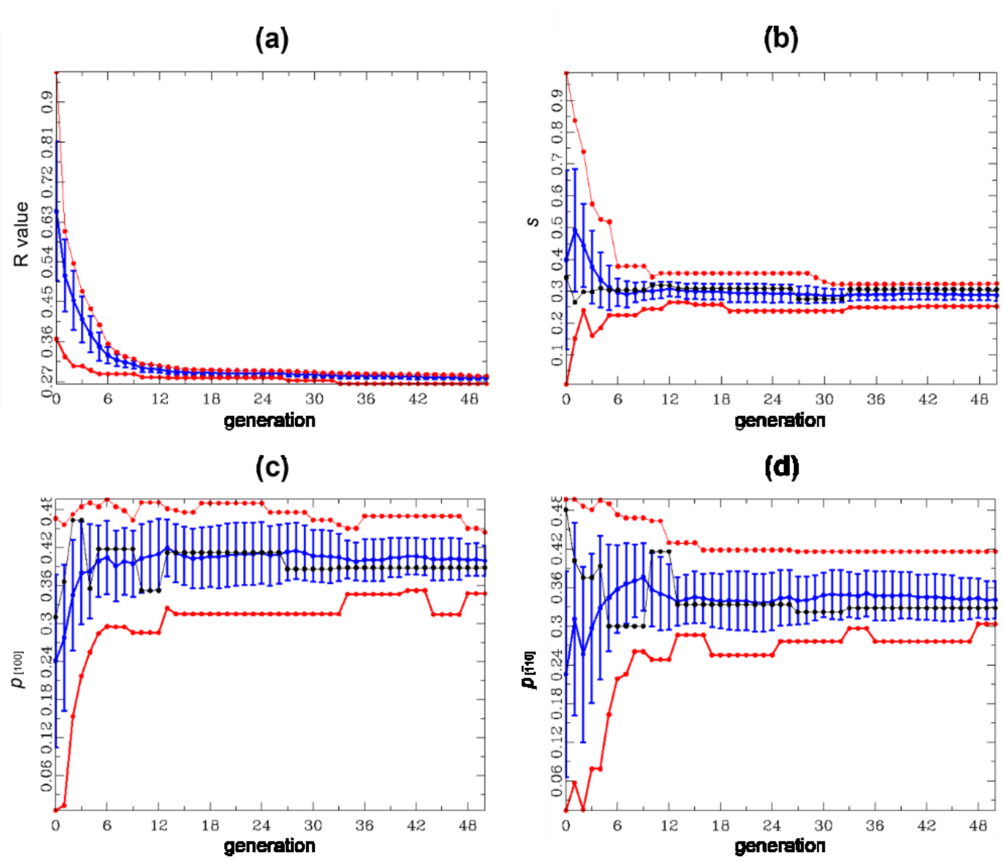

Figure S8 Refinement applied on the intensity profile of the diffuse streak indicated in the $[\bar{2}10]$ reciprocal space section in Fig. 6(b). Evolution of (a) the R value, (b) the stacking fault probability s, (c) the percentage $p_{[100]}$ of the $[100]$ twin and (d) the percentage $p_{[\bar{1}10]}$ of the $[\bar{1}10]$ twin as a function of the generation number. The figure shows the average (blue), smallest and highest (red) value at each generation. The value with the lowest R value at each generation is shown in black.
